# Revolutionizing bone regeneration: advanced biomaterials for healing compromised bone defects

**DOI:** 10.3389/fragi.2023.1217054

**Published:** 2023-07-14

**Authors:** Kamal Awad, Neelam Ahuja, Ahmed S. Yacoub, Leticia Brotto, Simon Young, Antonios Mikos, Pranesh Aswath, Venu Varanasi

**Affiliations:** ^1^ Bone Muscle Research Center, College of Nursing and Health Innovations, University of Texas at Arlington, Arlington, TX, United States; ^2^ Department of Materials Science and Engineering, College of Engineering, The University of Texas at Arlington, Arlington, TX, United States; ^3^ Department of Pharmaceutics and Pharmaceutical Technology, Faculty of Pharmacy, Future University in Egypt, Cairo, Egypt; ^4^ Katz Department of Oral and Maxillofacial Surgery, School of Dentistry, The University of Texas Health Science Center at Houston, Houston, TX, United States; ^5^ Center for Engineering Complex Tissues, Center for Excellence in Tissue Engineering, J.W. Cox Laboratory for Biomedical Engineering, Rice University, Houston, TX, United States

**Keywords:** craniofacial bone defects, oxidative stress, reactive oxygen species, semiconductors, biomaterials, engineered biomaterials

## Abstract

In this review, we explore the application of novel biomaterial-based therapies specifically targeted towards craniofacial bone defects. The repair and regeneration of critical sized bone defects in the craniofacial region requires the use of bioactive materials to stabilize and expedite the healing process. However, the existing clinical approaches face challenges in effectively treating complex craniofacial bone defects, including issues such as oxidative stress, inflammation, and soft tissue loss. Given that a significant portion of individuals affected by traumatic bone defects in the craniofacial area belong to the aging population, there is an urgent need for innovative biomaterials to address the declining rate of new bone formation associated with age-related changes in the skeletal system. This article emphasizes the importance of semiconductor industry-derived materials as a potential solution to combat oxidative stress and address the challenges associated with aging bone. Furthermore, we discuss various material and autologous treatment approaches, as well as *in vitro* and *in vivo* models used to investigate new therapeutic strategies in the context of craniofacial bone repair. By focusing on these aspects, we aim to shed light on the potential of advanced biomaterials to overcome the limitations of current treatments and pave the way for more effective and efficient therapeutic interventions for craniofacial bone defects.

## Introduction

In this review, we will discuss the current issues facing researchers in developing strategies to heal critical-sized and compromised bone defects. This includes understanding the role that large defects have on bone regeneration, complications associated with compromised healing, and aspects of aging and other related conditions that further confound this situation. We will also illustrate the role those new biomaterials must play as they are interventions to stabilize and promote bone defect healing. Further, we will discuss how these materials must play active roles in the healing process.

### Burden of care

**FIGURE 1:**
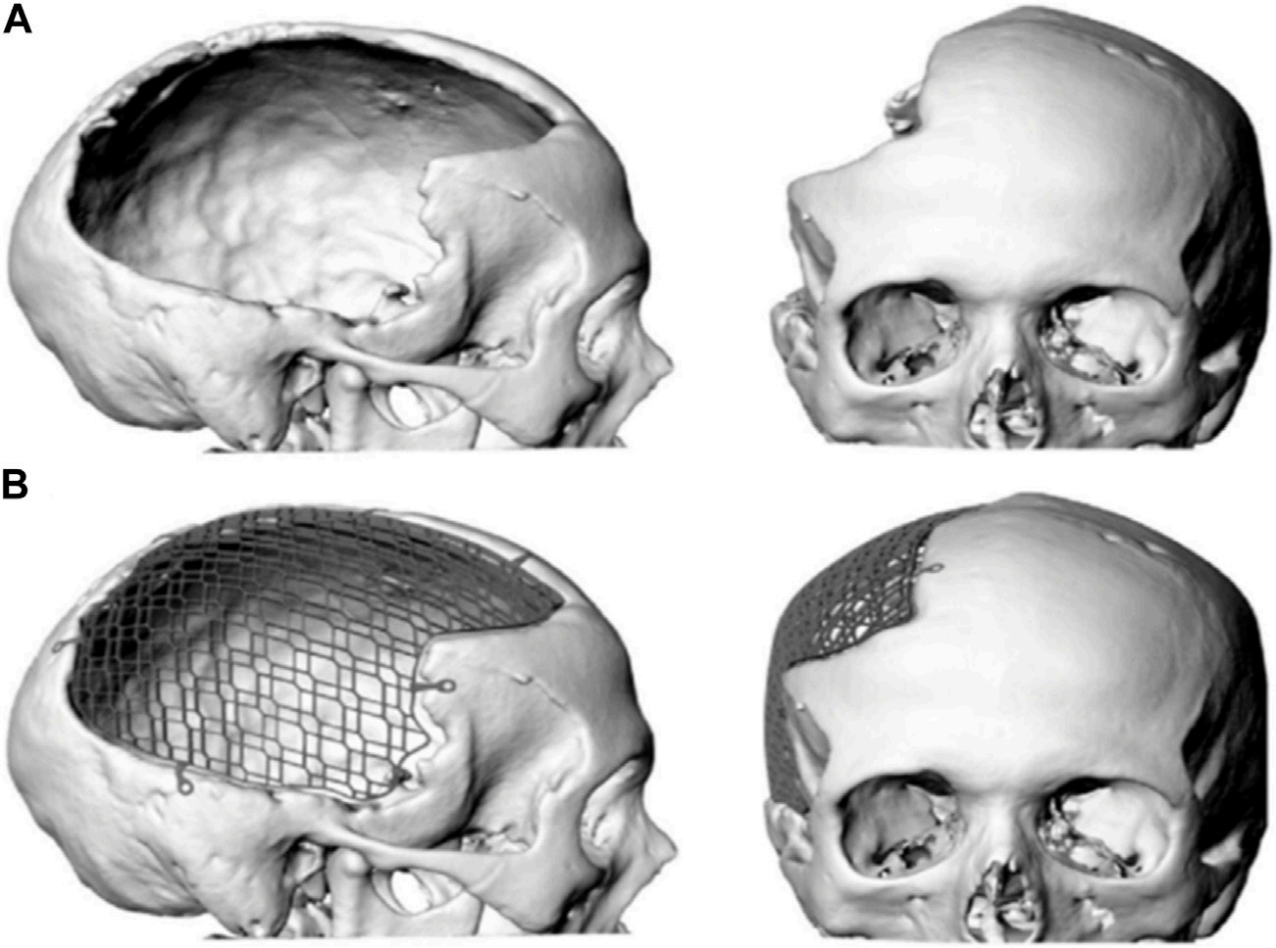
Digital image of a large cranial defect **(A)** with mesh implant **(B)**.

Large bone defects affecting the craniomaxillofacial region (Figure 1) (Koller et al., 2020) can arise from high energy impact, trauma, blast injuries, congenital bone defects, and the resection of locally aggressive tumors (Keating et al., 2005). A considerable number of these defects are sufficiently large and cannot spontaneously heal on their own. These defects are “critical-sized”, as they require surgical intervention and planned reconstruction to heal successfully. In all, more than 400,000 patients present to the emergency room with facial fractures or large craniofacial defects and require treatment each year, which costs over $1 billion in healthcare costs (Erdmann et al., 2008; Allareddy et al., 2011; American Society of Plastic Surgeons, 2017). Recent reports show that the incidence of craniomaxillofacial defects continues to rise by nearly 15% per year (Roden et al., 2012). Craniomaxillofacial defects, regardless of the cause, can affect function and esthetics which can be debilitating and socially incapacitating. Large-sized bone defects are also biomedically and economically burdensome (Szpalski et al., 2010). These defects require three-dimensional structural support, including permanent protection of the underlying brain tissue, mechanical integrity, allowance for the full range of jaw movement, and excellent facial esthetics along with faster healing rates.

### Complexity in craniomaxillofacial healing

The thickness and stiffness of cranial and maxillofacial bone varies according to different sites in the skull, and is covered with a multi-layered soft tissue envelope, making the craniomaxillofacial region a morphologically complex structure to heal (McElhaney et al., 1970; Dewey and Harley, 2021). As the skeleton ages, morphological changes occur. For example, in a study on skulls analyzed by dimensional changes in bone structure over time, the aged skulls exhibited resorbed bone in all areas (e.g., orbit, maxilla, mandible, skull) and likely led to soft tissue (e.g., muscle, fascia) laxity (Toledo Avelar et al., 2017). Another factor that affects the bone microenvironment is the various cell types. The vascular tubule-forming endothelial cells, bone-forming mesenchymal stem cells (MSCs), and osteoblasts and osteoclasts maintain bone homeostasis. The osteoblasts and osteoclasts work together for bone formation and bone resorption (bone remodeling) which maintains bone density and strength. The osteoblasts when mature, become incorporated in the bone matrix and become osteocytes. Osteocytes remain in the bone matrix and are responsible for bone turnover and adaptation (Aarden et al., 1994; Teitelbaum, 2007; Dewey and Harley, 2021). The endothelial cells also play an important role in homeostasis as they build and maintain vascular networks within the bone tissue. As we age, these homeostatic events become unbalanced, causing resorption of the existing bone structure coupled with soft tissue laxity or recession. This can cause bone to be more prone to defect formation or increase the potential of these defects to form upon injury, thereby increasing the burden of care. These bone defects may also be associated with compromised wound healing resulting from deficient vascularization, hypoxia, wound contamination, chemo/radiotherapy, or scarring from multiple surgical treatments. Studies have noted that compromised defects and injuries induced a delay in bone turnover rate (Sandukji et al., 2011; Hannemann et al., 2013) and re-vascularization in adults (Prasad and Bao, 2019) that imposed extended periods of hospital stay and significantly delayed healing time (Hwang and You, 2010). This delayed healing can be attributed to a marked increase in reactive oxygen species (ROS) (Sandukji et al., 2011), and prolonged inflammation (Wang et al., 2014). Prolonged oxidative stress causes damage to nucleic acids and proteins causing irreparable cellular injury and restricting cell viability, growth, and proliferation (Kubo et al., 2019). Furthermore, as we age, our bodies become less efficient at managing ROS and this can lead to oxidative stress. Oxidative stress occurs when there is an imbalance between the production of ROS and the body’s ability to eliminate them. Aging is also characterized by both impaired wound healing and chronic low-grade inflammation, also known as “Inflamm-Aging” (Ferrucci and Fabbri, 2018). Impaired wound healing can lead to a slower bone turnover rate, reduced re-vascularization, and prolonged inflammatory response, caused by an elevated level of ROS. Inflamm-Aging is caused by an increase in pro-inflammatory cytokines and a decrease in anti-inflammatory molecules due to factors like cellular damage, metabolic dysfunction, and the accumulation of senescent cells (Shafiq et al., 2021). Controlling inflammation could potentially delay the onset and progression of age-related diseases and improve overall health outcomes in older adults (Chung et al., 2019). To promote healing, it is necessary to increase tissue-level antioxidant activity to mitigate ROS and promote angiogenesis and osteogenesis.

Overall, intrinsic oxidative stress due to an underlying systemic disease can also impair bone regenerating capacity by the production of oxidants and elimination of protective antioxidant mechanisms (Sies, 1997; Hamada et al., 2009; Patel et al., 2013; Duryee et al., 2018; Li et al., 2020). Elevating tissue-level antioxidant activity can reduce ROS and promote angiogenesis and osteogenesis needed for healing (Wang et al., 2014). A controlled ROS level has a key role in the regulation of many fundamental cellular processes in the body such as proliferation, differentiation, and repair (Chavan et al., 2007; Mottaghi and Nasri, 2021). However, increased ROS production causes structural damage to the genomic DNA of osteoblasts and osteoclasts disrupting their normal function and can lead to apoptosis (Mlakar et al., 2010). ROS-activated lipid peroxidation-dependent lipoxygenase is associated with decreased osteoblastic activity and increased osteoclastic activity (Gullberg et al., 1997). ROS is a major determinant of oxidative stress and controls the remodeling capacity of bone (Jahanian et al., 2016).

### Role of antioxidant mechanisms in bone healing

Antioxidants play a key role in regulating a myriad of life processes within the body, including the musculoskeletal system. We often encounter antioxidants in our daily lives through the consumption of food, beverages, and taking supplements in our diets. These are dietary antioxidants which interface with our bodies by stimulating various cellular and extracellular matrix mechanisms that then couple to other mechanisms within cells. For example, a well-known antioxidant, Vitamin C or ascorbic acid, has been well documented to prevent many bone diseases and promote bone health (Rondanelli et al., 2021). Many of our food products contain several key vitamins and minerals to boost our antioxidant defenses. These defenses are mechanistic antioxidant enzymes expressed and secreted by tissues to reduce the accumulation of ROS or free radicals involved in the aging process. Mitochondrial ROS are highly reactive molecules produced as natural byproducts of cellular respiration within the mitochondria (Kageyama et al., 2021). While mitochondria are essential for generating energy in the form of ATP, they can also generate ROS during this process. Mitochondrial ROS includes molecules such as superoxide anions (O2•−), hydrogen peroxide (H_2_O_2_), and hydroxyl radicals (•OH). Although ROS are typically associated with cellular damage and oxidative stress, they also play important roles as signaling molecules in various cellular processes (Hernansanz-Agustín and Enríquez, 2021). In low levels, ROS can regulate cellular functions such as cell proliferation, apoptosis, and immune response. However, excessive accumulation of mitochondrial ROS can lead to oxidative damage of cellular components, including DNA, proteins, and lipids, contributing to the development of various diseases and aging processes (Hernansanz-Agustín and Enríquez, 2021; Kageyama et al., 2021). Therefore, maintaining a delicate balance of mitochondrial ROS is crucial for cellular health and overall wellbeing. Below we discuss how these mechanisms play a key role in bone signaling.

**FIGURE 2:**
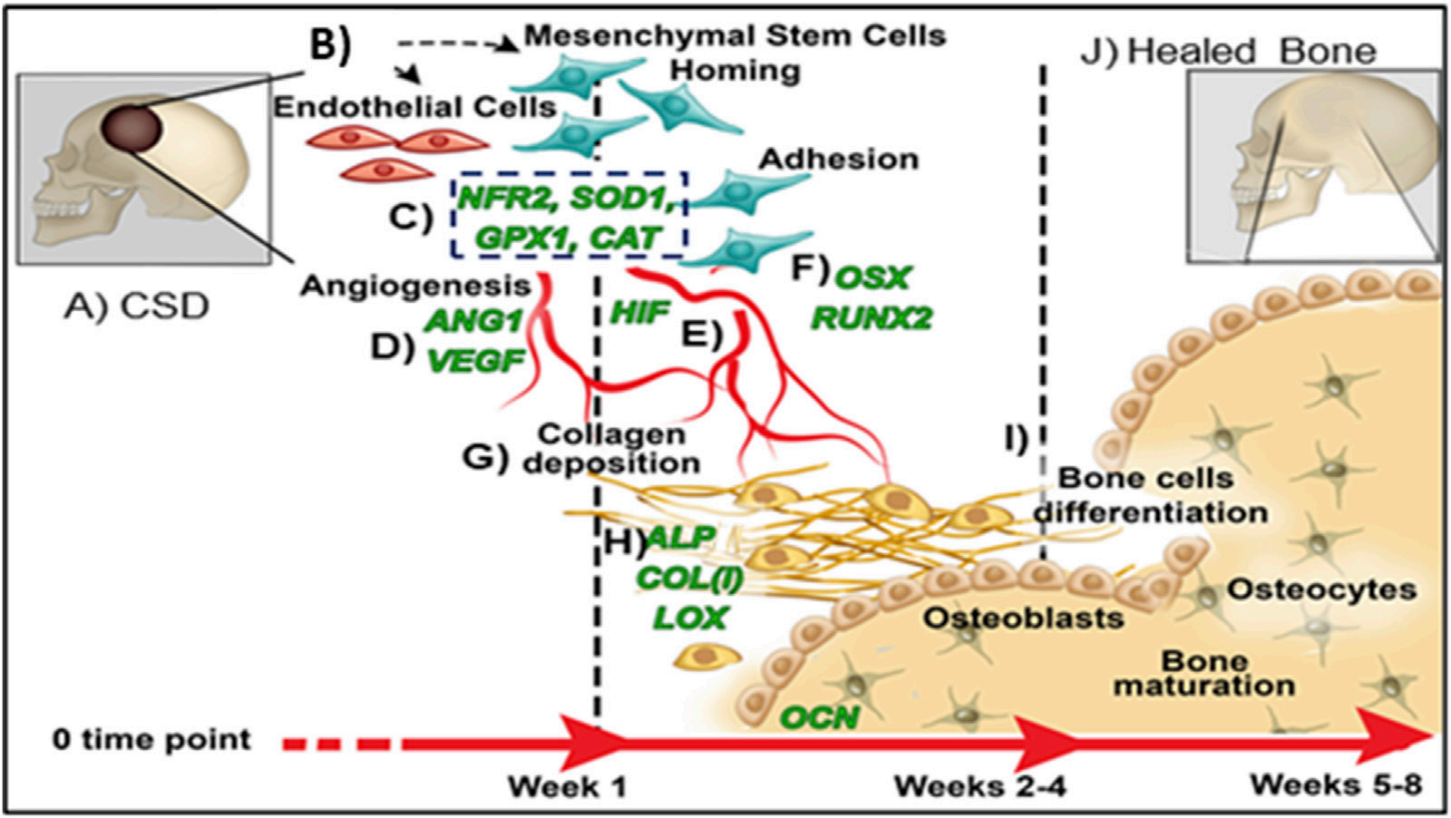
Proposed mechanism of healing CSDs over time: **(A)** Critical sized defect; **(B)** Endothelial Cells and Mesenchymal Stem Cells arrive in bone defect; **(C)** Antioxidant activity (NRF2, SOD1, GPX, CAT); **(D)** Angiogenic transcription markers expressed (HIF, ANG1, VEGF); **(E)** Vascular tubule formation; **(F)** Osteogenic transcription (RUNX2, OSX); **(G)** Collagen matrix formation; **(H)** Bone matrix protein synthesis (ALP, COL, OCN); **(I)** Bone formation; **(J)** Healed CSDs.

Antioxidant transcription factors such as nuclear factor erythroid 2-related factor 2 (NRF2) play a vital role in promoting bone and vascular healing (Figure 2) (Jakob et al., 2002; Lean et al., 2003; Mathy-Hartert et al., 2008; Yin et al., 2009; Sun Y.-X. et al., 2015; Kubo et al., 2019). NRF2 induces cell viability, migration, endothelial cell angiogenesis, and mesenchymal stem cell (MSC) osteogenesis (Sun Y.-X. et al., 2015) while acting as a master antioxidant promoter that reduces ROS to promote healing (Narimiya et al., 2019). NRF2 deficiency downregulates endothelial cell vascular endothelial growth factor (VEGF) expression, reduces bone strength by 30% and prevents bony union (Lippross et al., 2014). NRF2 is activated by two key mechanisms: 1) phosphorylation of cell surface glycogen synthase kinase- (p-GSK3-beta) that activates promoter regions on NRF2, and 2) the presence of electrophilic compounds such as sulforaphane can indirectly affect the Kelch-like ECH-associated protein (Keap1)-NRF2 nuclear binding through the modification of cysteine residues on the Keap1 surface which subsequently affect NRF2 (Dinkova-Kostova et al., 2005; Tebay et al., 2015; McMahon et al., 2018). This activation promotes downstream antioxidants (e.g., superoxide dismutase (**SOD1**) and glutathione peroxidase (**GPX1**)) (Mohammadzadeh et al., 2012; Ma, 2013; Sultan et al., 2018; Dai et al., 2020). SOD1 plays a vital role in osteogenesis (via collagen cross linking and osteocalcin expression (Badr, 2008; Nojiri et al., 2011; Sandukji et al., 2011; Yamada et al., 2013; Duryee et al., 2018)) while GPX1 promotes angiogenesis (via VEGF expression and vascular tissue repair (Galasso et al., 2006)). Thus, these key antioxidants signaling pathways are integral to the regeneration of new bone.

Several authors noted differences in NRF2-linked bone response for males and females as it relates to aging (Pellegrini et al., 2017). The bone phenotype of NRF2 moderate activation suggested sexual dimorphism. In male Keap1^+/−^mice, bone formation significantly increased, while bone resorption significantly reduced compared to their littermate controls. However, there were no notable effects observed in females (Han et al., 2022). Researchers noted sex differences in craniofacial healing, even considering the type of fracture or defect in bone (Merten et al., 2022). This was found in mouse models which showed that NRF2 activation through the disruption of Keap1 ubiquitination has some effects on bone mass (Yin et al., 2020). These differential effects indicate age and sex are factors affecting NRF2-linked bone response. This could help to explain key differences in bone morphology as we age. These appear in humans as morphological and mechanical changes which occur with changes in the frontal and peripheral craniofacial skeleton (Urban et al., 2016). However, since this field is still very new, extensive developmental or epigenetic studies in animals would need to be conducted to prove this hypothesis.

### Aging, oxidative stress, and compromised bone defects

As discussed above, aging offsets the balance in regenerating new bone and allows resorption of existing bone (Demontiero et al., 2012; Deng et al., 2022). This can cause small bone defects to heal improperly when under normal conditions they would heal on their own. For larger defects and fractures, this problem is exacerbated and can lead to permanent disability. For example, implant osteointegration is challenged due to alveolar and mandibular bone loss limiting their ability to replace missing dentition (Deng et al., 2022). During aging, alterations in local signaling result in a diminished ability of skeletal cell lineages to withstand stress. This leads to the promotion of a more fibroblastic phenotype, increased osteoclastogenesis, and pro-inflammatory cytokine production, along with decreased bone regeneration. These changes reflect the balance between skeletal homeostasis and regeneration (Tevlin et al., 2020). Aging affects bone differently from normal trauma-induced inflammation in that inflammation processes become less organized under aging conditions in which the NF-kB signaling pathway plays a key role (de Gonzalo-Calvo et al., 2010). Further, aged skeletal stromal cells showed decreased Wnt signaling, increased senescence, impaired cell function, and reduced osteogenic capacity that was partially rescued with administration of a dietary antioxidant resveratrol (Ambrosi et al., 2020; Clarke, 2021; Butler et al., 2022). For example, Wnt10b deficiency resulted in age-dependent loss of bone mass and progressive reduction in MSCs (Stevens et al., 2010). Also previous studies indicated that Wnt10b is significantly reduced with osteoporotic derived stem cells (Huang et al., 2023).

Oxidative stress is another condition limiting angiogenic and osteogenic activity. Oxidative stress induces loss of bone mass and strength and increased risk of fractures and impaired healing. Factors involved in elevated oxidative stress include estrogen deficiency, elevated endogenous glucocorticoid levels, age-related diseases, and prolonged exposure to ROS (Ardura et al., 2020). The exact mechanism perpetuating oxidative stress has not yet been elucidated, but the likely chemical induction mechanism could be related to increased ROS levels leading to cellular senescence (Liguori et al., 2018). Moreover, the loss of estrogens or androgens in the body weakens bone defense against oxidative stress and is responsible for an increase in bone resorption (Manolagas, 2010). Exacerbating this problem is the fact that inflammation and oxidative stress are inextricably linked to aging (Leyane et al., 2022), which can perpetuate the resorption of bone and throw off the homeostatic balance and regenerative capacity of bone to heal. Nature has developed various antioxidant mechanisms as defense against oxidative stress, but their efficacy decreases with aging (Portal-Núñez and Esbrit, 2013).

**FIGURE 3:**
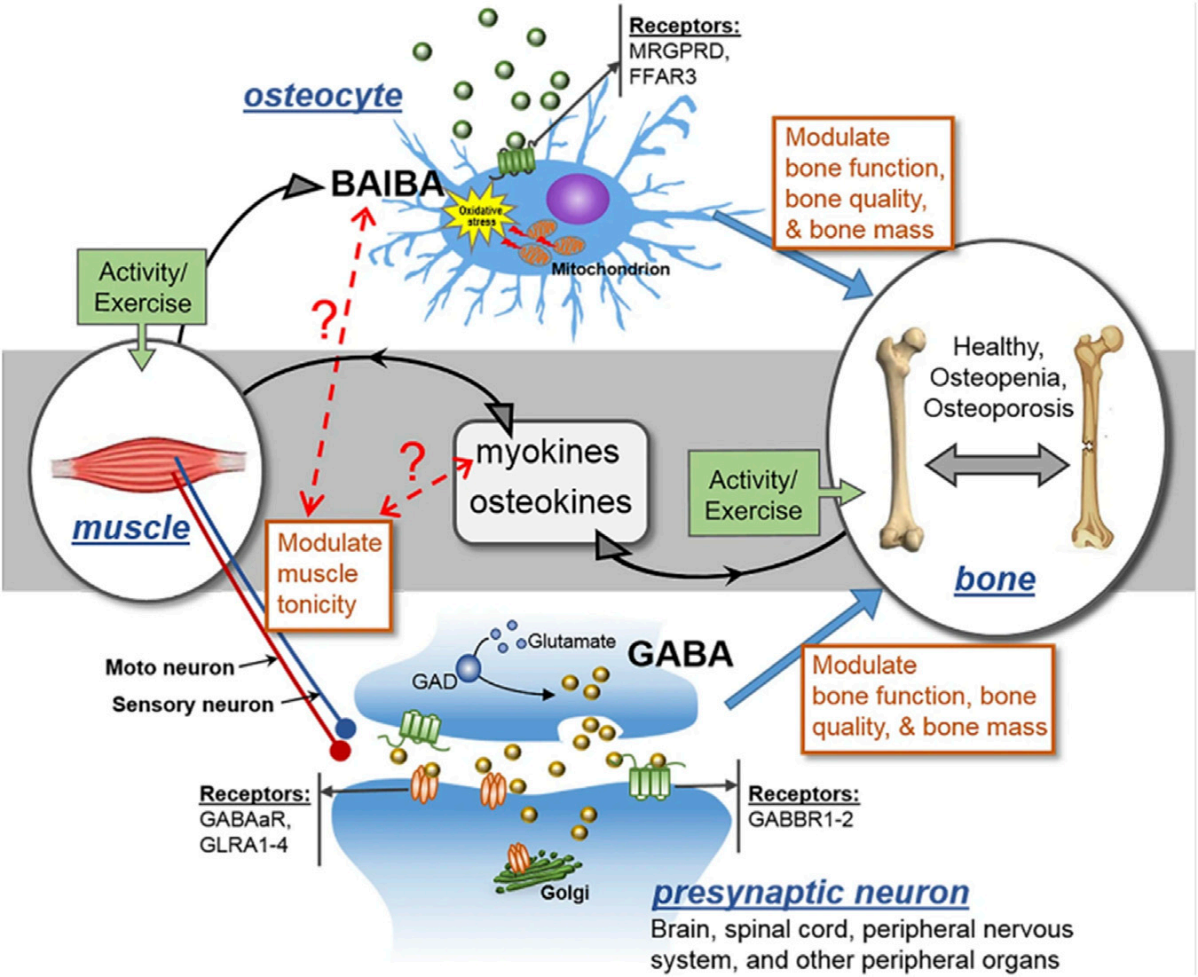
MRGPRD (MAS-related GPR family member D), FFAR3 (a G-protein-coupled receptor), GABAaR (gamma-aminobutyric acid type A receptor), GABBR1-2 (gamma-aminobutyric acid type B receptor subunit 1–2), GLRA1-4 (glycine receptor alpha 1–4), and GAD (glutamate decarboxylase) are all involved in the control of muscle tonicity. GABA is a major neurotransmitter that is generated in the central nervous system (CNS) and spinal cord, and its action controls muscle tonicity both centrally and peripherally. BAIBA is a myokine secreted from skeletal muscles that has direct effects on bone/osteocytes in mice. Exercise promotes the secretion of both myokines and osteokines, which can have autocrine and paracrine effects. It is hypothesized that muscle tonicity could potentially influence the release of myokines, which could in turn affect the levels of BAIBA, and *vice versa*. The receptors for GABA and BAIBA mediate their functions, and certain SNPs may act as modifiers of these effects. Thus, muscle tonicity may represent a novel mechanism for the regulation of myokine release and its effects on bone and muscle.

Several cell types occupying the skeletal framework are affected in diverse ways in aging or related conditions in bone. Oxidative stress induces negative effects on osteocytes (Kitase et al., 2018). ROS causes osteocyte apoptosis and cell death. L-Beta Amino Iso-Butyric Acid (L-BAIBA) reduces the impact of ROS on osteocytes (Wang et al., 2020). The protective effects of L-BAIBA are lost with age despite its production not being lost with age. The ability to activate oxidative stress protection in osteocytes is the main issue causing this phenomenon. The protective receptor involved in L-BAIBA protection involves activation of Mas-related G-protein-coupled receptor type D (MRGPRD, Figure 3) (Wang et al., 2020), MRGPRDs are regulators of bone homeostasis and reduced in expression as osteocytes and osteoblasts age (Hsiao et al., 2010; He et al., 2020). Thus, targeting these receptors could be used to mitigate the issues related to aging and promote bone homeostasis and regeneration.

Despite the benefits of antioxidants to fight aging or related conditions, dietary antioxidants do not have the capacity to heal large bone defects or fractures on their own. They are nutrients for every system in the body and to have these molecules locally delivered has become the subject of recent studies. Because of the large size of critical-size bone defects, they require the use of fixative or resorbable implants to stabilize the defect while facilitating bone regeneration. We will discuss the use of various biomaterials used to treat these defects and illustrate how these materials can target antioxidants to promote bone healing.

### Current biomaterial and tissue engineering treatment strategies

**FIGURE 4:**
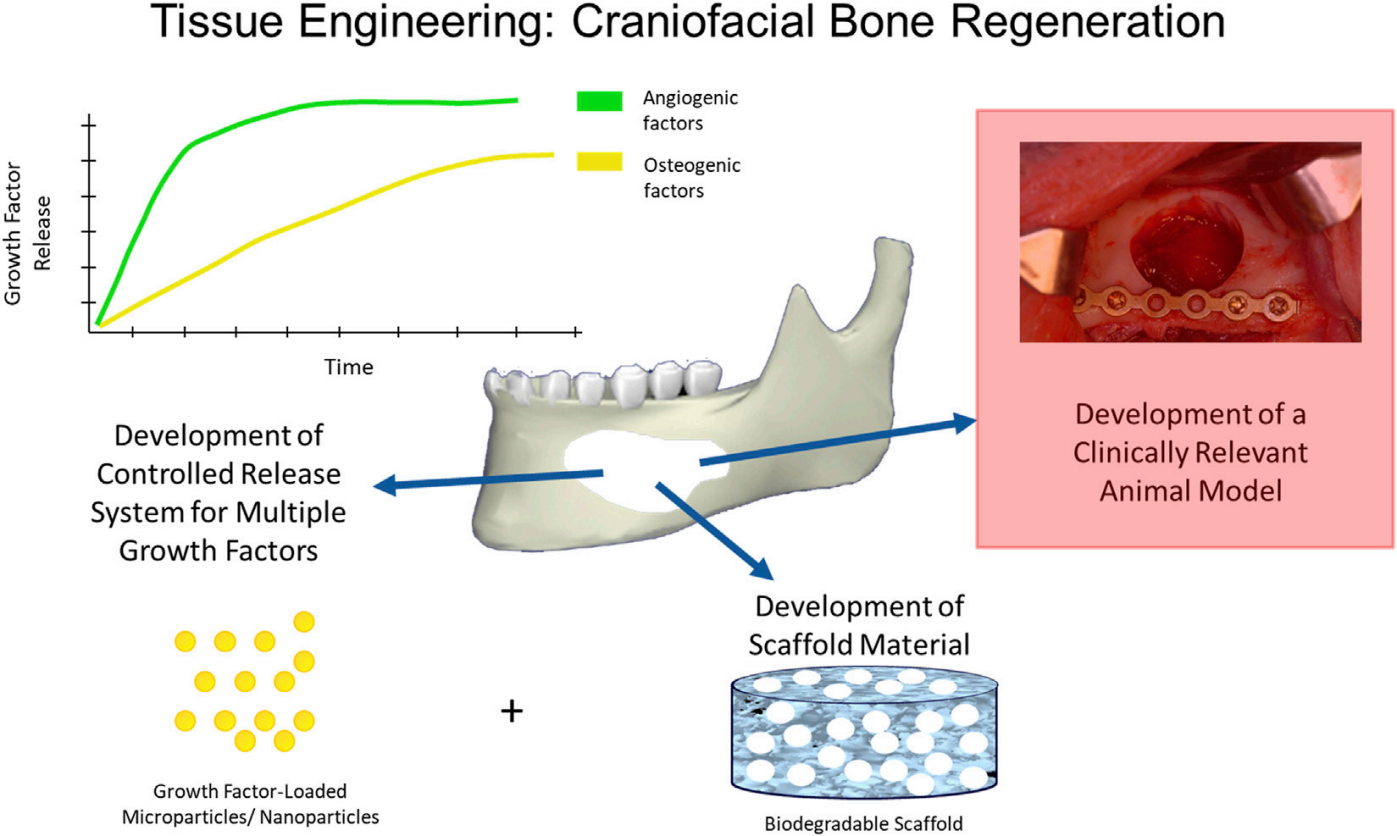
Tissue Engineering in Craniofacial Bone Regeneration. Many strategies have been used to induce bone regeneration in craniofacial defects and have employed various biomaterial formats (e.g., nanoparticles, scaffolds, implants) and/or bioactive factor release (e.g., small molecule, drug) to induce angiogenesis and osteogenesis for bone formation using clinically relevant animal models.

As mentioned above, large-sized craniofacial bone defects require three-dimensional structural support, including permanent protection to the underlying tissues, mechanical integrity, the support of full-range jaw movement, and facial esthetics along with faster healing rates, which makes them difficult to restore. Current treatment strategies for repair and reconstruction require a consideration of appropriate biomaterial scaffolds, bioactive factors, and appropriate delivery kinetics of those factors to the wound healing environment (Figure 4). Some of these are explained below.

## Autografts

Bone grafts from an autogenous source are the gold standard for treating craniomaxillofacial bone defects (Elsalanty and Genecov, 2009; Brown Baer et al., 2012; Kruijt Spanjer et al., 2017; Dewey and Harley, 2021). Autograft is bone tissue taken from a secondary site in the patient’s own body for replacing bone at the primary defect site. Iliac crest is one of the most common sites used for large autogenous bone grafts, with mandibular bone donor sites are commonly used when smaller volumes are needed. Smaller craniomaxillofacial defects show a very high success rate with autografts as it retains osteogenic and angiogenic cells and shows a favorable immunogenic response (Pogrel et al., 1997; Dewey and Harley, 2021). Despite the high success rate, there is limited bone availability for attaining autogenous bone grafts. Additionally, removing bone from a secondary area in the patient’s body can lead to an additional surgery site, pain, vascular and nerve injury, secondary bone fracture, high chances of bone morbidity and longer healing times (Elsalanty and Genecov, 2009; Dewey and Harley, 2021).

## Allografts and xenografts

Allografts are bone grafts taken from a donor of the same species and xenografts are bone grafts taken from a different species. These grafts overcome drawbacks of autografts such as the need for secondary donor-site surgery, and increased chances of bone morbidity. Allografts and xenografts are required to undergo a series of processing such as decellularization and demineralization to minimize the immunologic response and disease transmission (Elsalanty and Genecov, 2009; Dewey and Harley, 2021). The vigorous pre-processing of these grafts can lead to a less osteogenic biomaterial, as it affects the extracellular matrix and collagen in the donor bone (Bae et al., 2006; Ghanaati et al., 2014; Dewey and Harley, 2021). There can also be significant variability between the different bone tissues that are being processed. Allograft and xenograft, even after sterilization, can lead to post-operative infections and unfavorable immunologic reactions. The bone grafts also have low mechanical strength for long-term stability in cases of large bone defects.

## Fixative metals and coatings

Large and complex craniofacial bone defects often require reduction of mobile segments, fixation using metal devices, and volume-filling bone substitutes to stabilize and regenerate the lost bone. Titanium (Ti) fixative devices or mesh provide strength to support cranial defects. Yet, they are unable to speed healing rates due to a lack of bioactivity (Iwai-Yoshida et al., 2012) or aseptic loosening (Jokstad, 2004; Katz et al., 2007; Gita and Ravi, 2011; MacInnes et al., 2012). Attempts to coat these fixation plates with bioactive hydroxyapatite (HA) or silica based Bioglass™ (45S5) did not improve bone regeneration rates. Coating structural quality suffered from high temperature processing (enameling, plasma-spraying) that induced metal-coating thermal expansion mismatch and interfacial cracking (Thomas et al., 1987; Hayashi et al., 1989; Soballe et al., 1990; Dhert et al., 1991; Klein et al., 1991; Wang et al., 1993; Yang et al., 1996; Bloyer et al., 1999; Foppiano et al., 2007), reduced quality and bioactivity owed to glass crystallization and mixing of soluble and insoluble phases (Koeneman et al., 1990; Ji et al., 1992; Weinlaender et al., 1992; Filiaggi et al., 1993; Frayssinet et al., 1993; Zyman et al., 1994; Mcpherson et al., 1995; Gross et al., 1997; Gomez-Vega et al., 2000; Gomez-Vega et al., 2001; Oku et al., 2001). As a result of these factors, immature bone healing and fibrous tissue attachment were observed in canines after 12 weeks (Oirschot et al., 2014) with poor long-term healing (Roy et al., 2011). As defect filler, mesoporous 45S5 fully resorbed, but only had 32%–38% of defect healing in 3 months (Liu et al., 2013; Zhao et al., 2015; Zhang et al., 2016).

## Ceramics

Clinicians use ceramics and hydroxyapatite-based materials in dental clinics. Bioglass and tricalcium phosphate are alternatives to allografts and autografts for bone regeneration (Athanasiou et al., 2010; Broggini et al., 2015; Tatara et al., 2019; Dewey and Harley, 2021). These calcium- and phosphorus-containing bioceramics have good biocompatibility and acceptable mechanical properties for defect stability. However, these materials are not as successful as bone grafts due to their brittle nature, longer resorption times and higher infection rates (Dewey and Harley, 2021). Other ceramic materials such as nanosilicates and silicate nanoparticles have also been studied and show promising results in combination with metals and other biomaterials (Dewey and Harley, 2021). Many clinicians use silica (SiO_2_) based biomaterials for their antibacterial properties (Ferraris and Spriano, 2016; Ferraris et al., 2021). Yet, silica-/bioactive glass-based nanoparticles and surface modifications (Gomez-Vega et al., 2000; Sanchez et al., 2005; Chen et al., 2011; Catauro et al., 2015) have not adequately shown any antioxidant effects.

## Polymers

Many researchers have studied natural polymers derived from animals and plants (i.e., collagen-based biopolymers) for their effect on soft and hard tissue healing. These polymers have tunable porosity and orientation which is beneficial for use in drug delivery applications. However, due to the poor mechanical properties, there has been limited applications for these biomaterials. Synthetic polymers such as polycaprolactone (PCL) and poly (lactic acid) (PLA) are FDA approved for tissue engineering applications. They are biodegradable, biocompatible and have tunable biomechanical properties (Athanasiou et al., 1996; Gredes et al., 2016; Dewey and Harley, 2021). However, the synthetic biopolymers have longer than expected degradation rates and can produce cytotoxic degradation products.

## Nano-composite resorbable materials

For bone substitutes, autogenous grafts are the gold standard due to limited immune response and endogenous cells, yet, donor site morbidity and low secondary site volume limit their use (Chatterjea et al., 2010). Collagen scaffolds produce new bone (modulus = 10 GPa, close to existing bone) in rat cranial CSDs, yet even with MSC inclusion, only 39% of the defect healed after 10 weeks (7.02 mm^3^ new bone volume/18.09 mm^3^ total defect volume (%BV/TV) (Al-Hezaimi et al., 2016). Additionally, extended culture time required for obtaining high cell numbers, exposure to serum prions and peptides, reduced viability, and increased senescence can contribute to the rejection of MSCs (Undale et al., 2009). Gelatin or chitosan hydrogels promote cell growth, form a glycosaminoglycan-like structure, and degrade (Angele et al., 2009; Chung and Burdick, 2009; An et al., 2010; Ragetly et al., 2010; Bian et al., 2013; Kim et al., 2013; Salamon et al., 2014; Xavier et al., 2015), yet they need modification to structurally support defects for new bone formation. Biopolymer surfaces modified with single peptides lack multi-functionality to mimic extracellular matrix (ECM) (Collier and Segura, 2011) and have a short half-life (Chow et al., 2008) while mini-proteins limit angiogenesis via low vascular endothelial growth factor (VEGF) activity and no antioxidant effect limiting their use (Treggiari et al., 2015). Recombinant human bone morphogenic protein (rhBMP2) (1–10 mg dose (Zara et al., 2011; Brierly et al., 2016), released by collagen scaffold) can have severe side effects including ectopic bone growth, prolonged inflammation, soft tissue swelling at the surgical site, cyst-like bone growth *in vivo*, and only 15% higher healing vs bare collagen scaffolds after 4 weeks due to rapid rhBMP2 depletion (Carragee et al., 2011; Zara et al., 2011; Lee et al., 2012; Brierly et al., 2016; Song et al., 2016; Shi et al., 2017; Ramly et al., 2019).

### Limitations of biomaterials and antioxidant treatments for bone injuries

As discussed above, NRF2 is a key transcriptional factor that is responsible for activating an antioxidant response reaction against oxidative stress (Taguchi et al., 2011). NRF2 has also been known to affecting bone healing rates by maintaining homeostasis in bone cells, suggesting that NRF2 can promote fracture healing in the presence and absence of oxidative stress, thereby implicating its role in bone healing after traumatic injury (Sun Y. X. et al., 2015; Kubo et al., 2019). When there is no fracture injury or bone defect present, Keap1 (Kelch-like erythroid cell-derived protein with cap ‘n' collar homology-associated protein), a cytoplasmic antagonist, negatively regulates NRF2, resulting in the ubiquitination of NRF2 and its degradation by the ubiquitin proteasome system (UPS) (Wruck et al., 2008; Canning et al., 2015). Upon injury, NRF2 is mobilized by Keap1 cytosol transport into the nucleus, chelation with cations, and release of NRF2 to activate downstream antioxidant reactive elements. This then activates a downstream cascade of osteogenic and angiogenic transcription to stimulate bone formation.

Patients given exogenous or dietary antioxidants that target these endogenous antioxidant mechanisms such as Vitamin E, Vitamin C, carotenoids, and polyphenols improved overall bone health. Patients given dietary or natural antioxidants after fracture had higher SOD1 activity, which reduced ROS and increased osteocalcin activity (Badr, 2008; Sandukji et al., 2011), and lowered healthcare costs by lowering hospital stays (Fabian et al., 2011). Antioxidants exert these beneficial effects by electrochemically reducing ROS via increased antioxidant activity (e.g., NRF2, SOD1, and glutathione peroxidase (GPX)) (Lean et al., 2003; Mathy-Hartert et al., 2008) and increasing the cation concentration thereby increasing the overall cations available for ROS reduction (Lü et al., 2010). This leads to prompt healing in compromised defects by limiting inflammation and decreasing patient recovery time (Simunovic et al., 2011).

In some cases, though, exogenous factors exhibited limitations in efficacy for osteogenic and angiogenic activity with associated side effects (Fabian et al., 2011). Exogenous factors such as drugs (e.g., nitrates) or gene therapy vehicles (e.g., viral vectors) resulted in poor endogenous antioxidant activity, impeded endothelial cell function due to cytotoxicity, immune toxicity, nitrate tolerance, and altered gene expression (Duvall, 2005; Papageorgiou et al., 2013; Sun Y.-X. et al., 2015; Daiber et al., 2017). Resveratrol (<2,000 mg/d) and N-acetyl cysteine (NAC <30 mM) are potent drugs that promote NRF2 and SOD1 expression (Ungvari et al., 2010; Ali et al., 2016). However, they can cause nausea or diarrhea at higher doses (Schmidt and Dalhoff, 2001; Salehi et al., 2018) and only slightly improved healing rates (25% BV/TV ^51^) or slightly reduced defect size (30%, 30 days (Casarin et al., 2014)) in small defects (<6 mm diameter) *in vivo*. Thus, for targeted approaches, local delivery may be the alternative option to maximally affect large bone defect healing. Further, biomaterial strategies that incorporate fixation devices and/or biopolymer scaffolds will play a larger role in stabilizing and healing these large defects due to the large volume of missing bone. We discuss these strategies below.

### Semiconductor materials for bone healing

**FIGURE 5:**
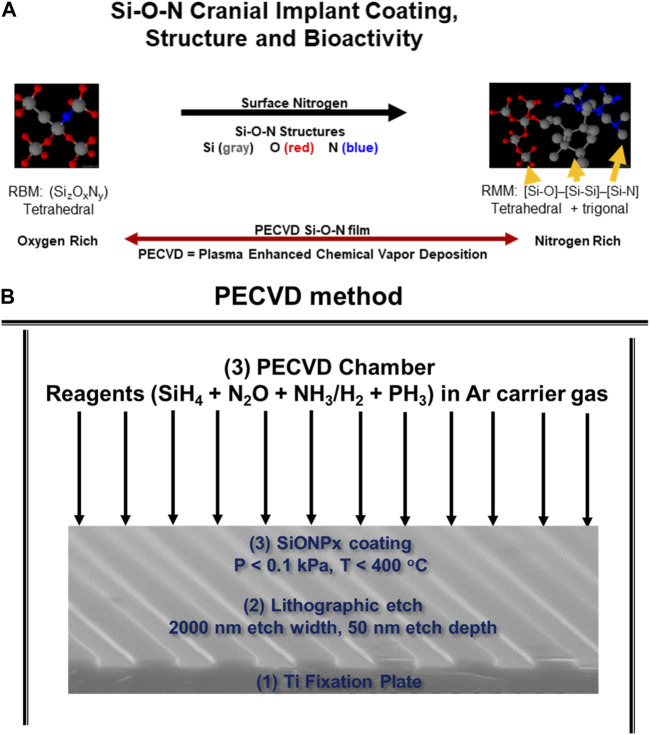
**(A)** Schematic of PECVD process to form SiONx coatings for Ti implants. **(B)** Surface formation of hydroxyl, phosphate, and carbonate groups that make up bone mineral hydroxyapatite when introduced to *in vitro* environment.

As we discussed above, fixation devices stabilize bone defects but cannot directly contribute to rapid bone healing due to their bioinert nature and subsequent inability to activate antioxidant mechanisms. Coatings could be used to solve this issue. Such coatings must adhere well to the underlying Ti while also stimulating antioxidant activity via electron or covalent structure on the coating surface. Materials such as dielectric coatings provide semiconductors with rigorous and reproducible performance by controlling computers in the nano-electronics industry. The dielectric coating on the surface of a semiconductor that controls the release of current into an integrated circuit is fabricated by a process known as plasma-enhanced chemical vapor deposition (PECVD, Figure 5). This method deposits thin films on substrates from a gas (vapor) state to a solid state. The PECVD technique uses plasma, instead of thermal energy in conventional CVD (Duryee et al., 2018). This method creates a surface with amides and hydroxyl groups, forms an amorphous coating, and helps in evenly sputtering of the molecules which can maintain the surface morphology of the substrate (e.g., implant). Further, the coatings are fabricated at relatively low temperature (<400°C), thus, thermal expansion mismatch between the implant substrate and coating layers is markedly reduced (Gredes et al., 2016). The PECVD process is advantageous as the coating thickness, atom ratio, and interfacial formation onto a biomedical device are under computer control. Furthermore, the thin films are formed relatively quickly (within 1 h), and have high reliability and repeatability in manufacturing (Gredes et al., 2016).

**FIGURE 6:**
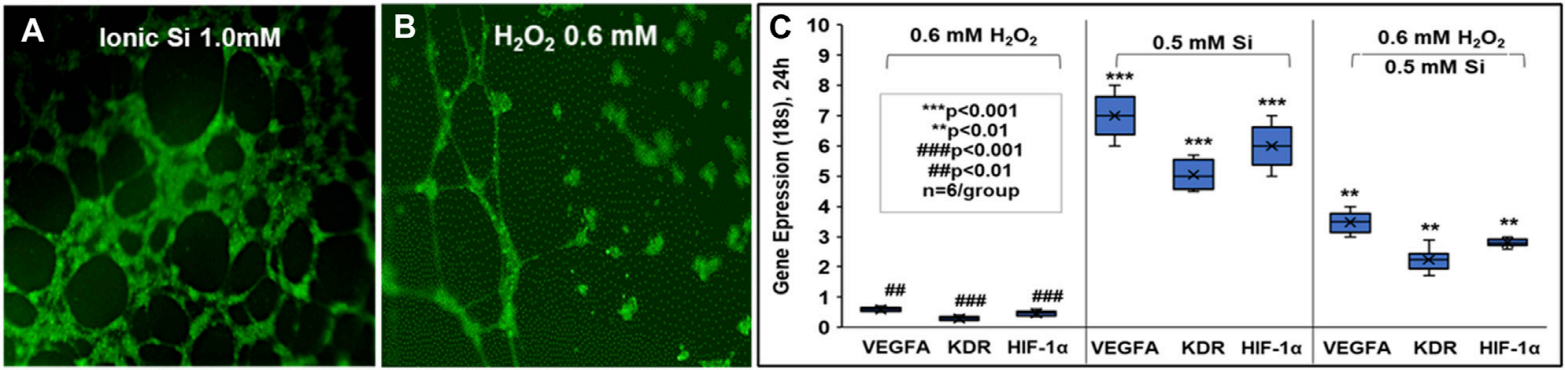
Effect of small molecular delivery of Si^4+^ that rescues human endothelial cell angiogenesis when exposed to reactive oxygen species and normal conditions. Primary human endothelial cells showed thick and dense tubules when exposed to 1.0 mM ionic Si **(A)** vs no Si treatment exhibiting immature tubule formation **(B)** in 24 h *in vitro*. HUVECs under ROS conditions (H_2_O_2_) showed increased angiogenic marker expression vs no Si ion treatments **(C)** (All experiments were performed with n = 6 per group according to protocols and methods published by Monte et al. (Monte et al., 2018).

**FIGURE 7:**
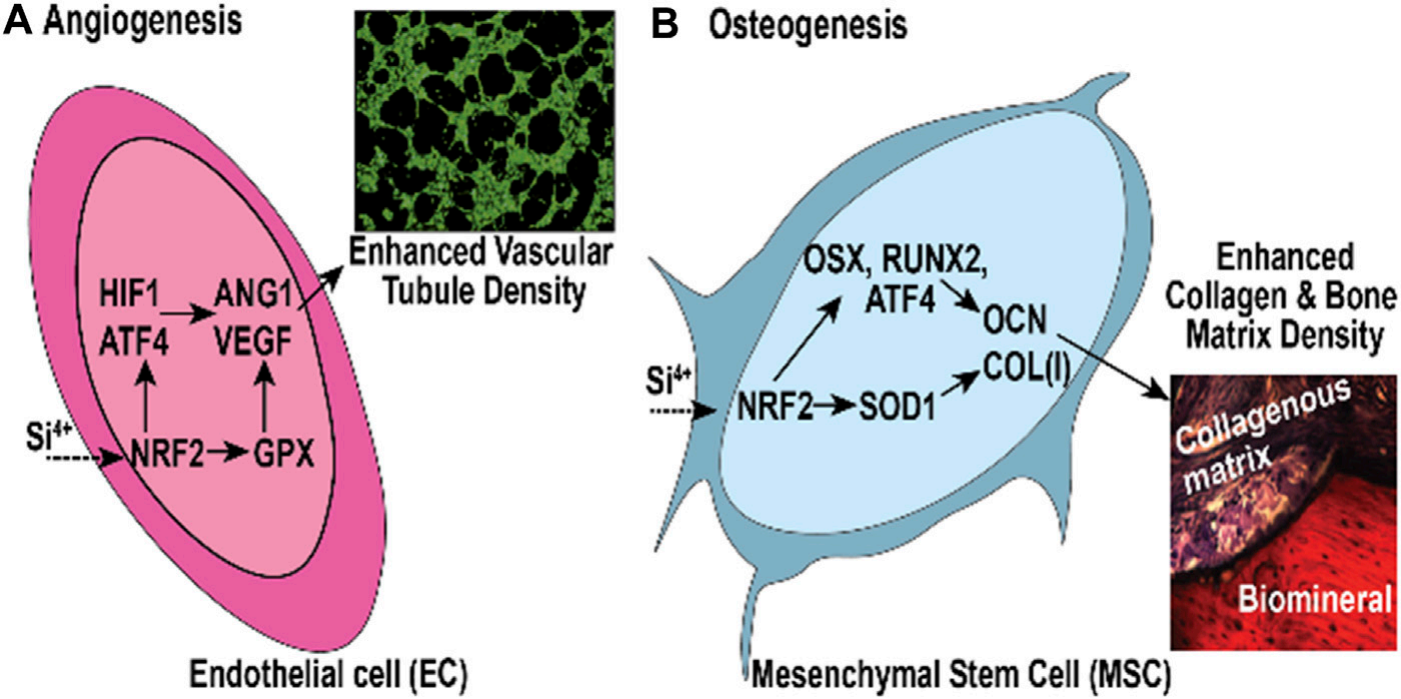
*In Vitro* and *In Vivo* model of NRF2 effect on **(A)** angiogenesis and **(B)** osteogenesis in bone regeneration.

We apply these concepts to bone regeneration via the material coating’s ability to sustain the release of degradation products which have a positive antioxidant effect and enhanced *in vitro* osteogenic biomarker expression. We fabricated silicon oxynitride (SiONx) coatings for implant surfaces using PECVD (Ilyas et al., 2015; Varanasi et al., 2017; do Monte et al., 2021). The PECVD process led to a stable coating of amorphous silica on the substrate material and released Si^4+^ for several weeks (Ilyas et al., 2016). This was accomplished by optimizing the surface nitrogen-to-oxygen (N/O) ratio in the coatings. This is an additional advantage of using PECVD for biomedical devices (Monte et al., 2019; Awad et al., 2022). For example, the nitrogen-to-oxygen atom ratio is controlled by controlling the source gases NH_3_ and N_2_O under the reductive ionized gas environment. This leads to varying levels of tetrahedral and trigonal chemical bond structure depending on the N/O atom ratio (Figure 5) (Varanasi et al., 2017) within the films which can change the surface charge, change the Si^4+^ ion release rate, and change the subsequent antioxidant response by cells. In fact, these implant coatings stimulated SOD1 activity and formed surface hydroxyapatite (HA) leading to osteogenesis in mouse osteoblast cells (Ilyas et al., 2015; Ilyas et al., 2016; Varanasi et al., 2017; Ilyas et al., 2019). Further, these coatings and their release of Si^4+^ appeared to rescue angiogenic activity in the presence of ROS in human endothelial cells (Monte et al., 2019). This shows the benefits of these coatings to play an antioxidant role during bone healing. Previous studies in our lab show that SiONx and Si^4+^ reduce ROS through cationic reduction, endothelial cell activity (Figure 6) (Monte et al., 2018), and enhanced SOD1 activity while enhancing proliferation and differentiation of osteoprogenitor cells (Awad et al., 2019; Ahuja et al., 2021). Furthermore, these biomaterials have been tested on skeletal muscle cells and showed antioxidant activity as indicated by attenuating the toxic oxidative stress induced by hydrogen peroxide (Awad et al., 2021). Our previous study on the effect of Si-ions on C2C12 myoblast cells showed that Si-ions exhibit significant antioxidant properties and can mitigate oxidative damage in these cells as shown from the significant increase of NRF2 and SOD1 gene expressions (Awad et al., 2021). Furthermore, our recent studies indicated that cells treated with H_2_O_2_ induce a significant increase in ROS production (Detected by Intracellular ROS fluorescent dye) compared to the normal control group with a *p*-value of 0.0006, while treating the cells with Si-ions significantly decrease the ROS production under H_2_O_2_ conditions (*p*-value = 0.0003 compared to H_2_O_2_ negative control group, data is not shown). When co-treated with different Silicon ion concentrations, ROS production significantly decreased compared to the H2O2 group Thus, the premise of our work is that oxidative stress, inflammation, and defect instability act as a large barrier for the rapid healing of severe bone loss. In contrast, SiONx-based coatings on fixative devices that release Si^4+^ will overcome this barrier by providing structural stability while inducing optimal antioxidant activity to lower ROS, and inflammation, and increase angiogenic and osteogenic activity to promote rapid defect healing (Figure 7).

### Animals models to test normal and compromised tissue healing in bone

Many of the models of bone healing and approaches have examined the use of new treatment modalities in healthy bone. Several animal models have been utilized to study the bone regeneration after induced bone defects such as rats (do Monte et al., 2021; Ilyas et al., 2016), rabbits (Shah et al., 2016; Piotrowski et al., 2019), pigs (DeMitchell-Rodriguez, 2023), and dogs (Taha et al., 2023). A prime example of a rabbit model of tissue regeneration would be a critical sized defect model in which an 8–10 mm diameter trephine defect is administered in the body of the mandible. A titanium plate is then secured along the inferior side of the mandible to prevent iatrogenic fracture (Shah et al., 2016). In such a model, several types of interventions can be studied from implant coatings to dietary effects to implantable scaffolds.

**FIGURE 8:**
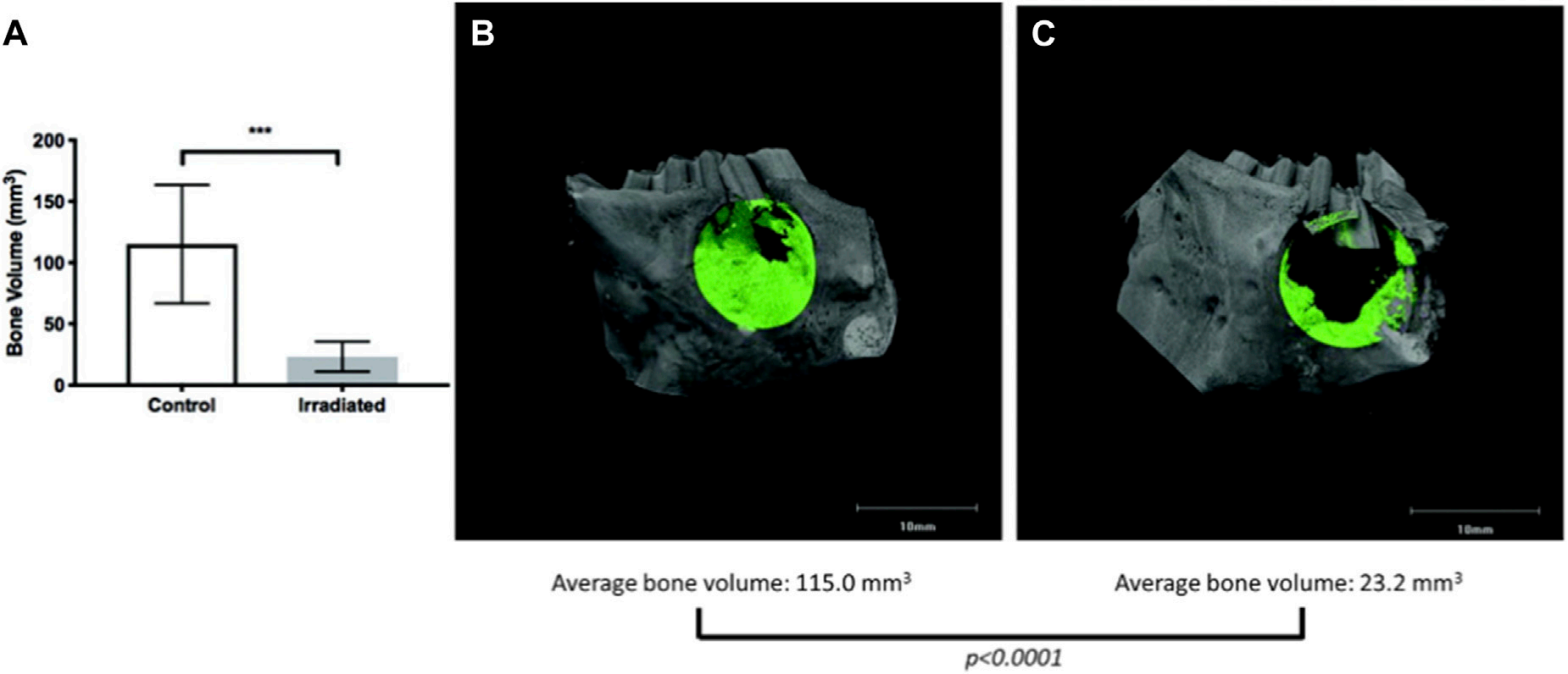
Micro-CT of compromised wound healing environment shows decreased bone formation in targeted area **(A)** Quantification of bone volume **(B)** Bone healing in control vs **(C)** irradiated animal defect and bone healing.

**FIGURE 9:**
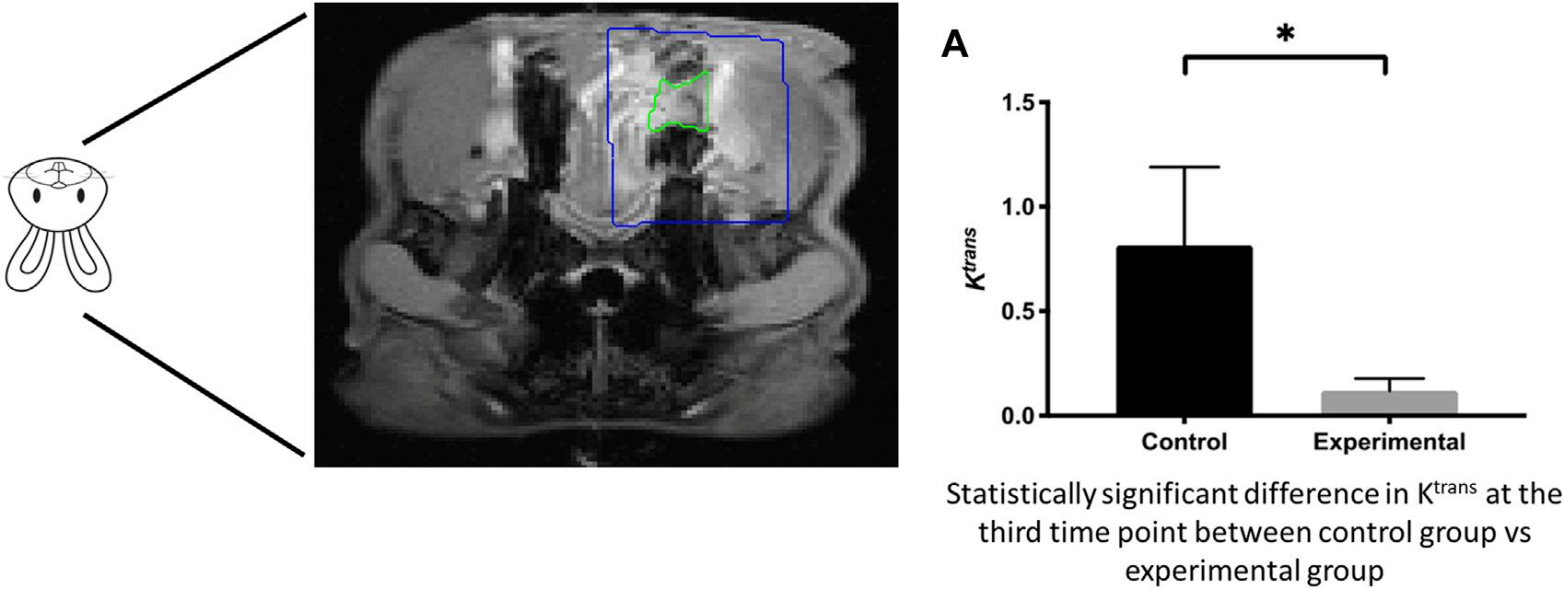
Compromised healing model of bone regeneration. DCE-MRI of compromised wound healing environment shows decreased tissue perfusion in targeted area.

However, few have studied bone regeneration strategies in the setting of a compromised wound. Preclinical models exhibiting a compromised wound healing environment are ones in which an initial insult to the bone is performed with either radiation or medications (i.e., bisphosphonates), mimicking osteoradionecrosis or medication-related osteonecrosis of the jaw, respectively. Preclinical animal models by Young and others (Piotrowski et al., 2019; Piotrowski et al., 2020) have studied aspects of compromised tissue environments. The basic premise is that the degenerating conditions afflicting bone act as a large barrier to inducing the natural healing processes of bone (Figure 8) (Piotrowski et al., 2019) and (Figure 9) (Piotrowski et al., 2020). This is a translational animal model that mimics the morbidities faced by patients suffering from osteoradionecrosis. Because the condition involves initial radiation of the animal, the natural response by these animals is a compromised state in which many of the normal functions of bone homeostasis and healing are impaired due to the insult to the bone cell microenvironment. Thus, these animal models could be potential candidates to study the conditions placed upon healing.

## Summary and future work

As discussed above, severe bone injuries are challenging to heal and reconstruct. Exacerbating this issue and complicating the healing process is the high prevalence of these patients afflicted with aging conditions. The use of traditional materials that were normally used to reconstruct the skeleton have low efficacy in stabilizing the bone layer if the disease or disorder continues to weaken the bone structure. Thus, treatment strategies and manufacturing of devices must incorporate new methods and materials to handle these conditions as well as stabilize the bone layer. The need for these new treatment strategies for targeting mechanisms involved in countering the aging condition while also stimulating faster regeneration of these bone structures will be the future development direction for healing these injuries. The use of improved methods of manufacture and materials with intrinsic properties or release of small molecules or drugs to target aging mechanisms to regulate cellular aging will be key to improving the outcome for patients and meet the burden of care. Still, these studies focusing on one approach yielded highly differential outcomes such that the clinical need cannot be met. Further, due to the complementary nature of the clinical need of fixation devices and bone substitutes to treat large bone defects, there is a need for new classes of biomaterials with similar compositional constructs. This will yield more predictable bone regeneration of biomaterials for sustainable and beneficial outcomes.
